# Hepatitis C Virus Genotype Diversity among Intravenous Drug Users in Yunnan Province, Southwestern China

**DOI:** 10.1371/journal.pone.0082598

**Published:** 2013-12-17

**Authors:** Zhihui Zhang, Yufeng Yao, Wenlong Wu, Ruilin Feng, Zhongxiang Wu, Wei Cun, Shaozhong Dong

**Affiliations:** 1 Yunnan Key Laboratory of Vaccine Research & Development on Severe Infectious Diseases, Yunnan Engineering Research Center of Vaccine Research & Development on Severe Infectious Diseases, Institute of Medical Biology, Chinese Academy of Medical Sciences & Peking Union Medical College, Kunming, Yunnan, China; 2 HIV Confirmatory Laboratory, Kunming Center for Disease Control and Prevention, Kunming, Yunnan, China; Centers for Disease Control and Prevention, United States of America

## Abstract

**Background:**

Recently, high proportions (15.6%–98.7%) of intravenous drug users (IDUs) in China were found to be positive for hepatitis C virus (HCV). Yunnan Province is located in southwestern China and borders one of the world's most important opium-producing regions, thus it is an important drug trafficking route to other regions of China.

**Methodology/Principal Findings:**

Here, we assessed 100 HCV-positive plasma samples from IDUs who were enrolled through the Kunming Center for Disease Control and Prevention in 2012. HCV C/E1 fragments were PCR-amplified and sequenced. We identified eight HCV subtypes (1a, 1b, 3a, 3b, 6a, 6n, 6u and 6v), of which genotype 6 was most predominant (frequency, 47%) followed by genotypes 3 (41%) and 1 (12%). HCV subtypes 6n (30%) and 3b (29%) were most common and were identified in 59% of the IDUs. We compared HCV genotypes among IDUs in Yunnan Province with those from other regions and found that the distribution patterns of HCV genotypes in Yunnan Province were similar to those in southern China, but different from those in eastern China. However, the distribution patterns of HCV subtypes varied among Yunnan Province and southern China, despite the shared similar genotypes. A comparison of the current data with those previously reported showed that the frequency of HCV genotype 6 increased from 25% to 47% within 5 years, especially subtypes 6a (5% to 15%) and 6n (11.2% to 30%). In contrast, the frequencies of subtypes 3b and 1b decreased by almost 50% within 5 years.

**Conclusion/Significance:**

Our results provided further information to support the assertion that drug trafficking routes influence HCV transmission patterns among IDUs in Yunnan Province. The frequency of HCV genotypes and subtypes changed rapidly among IDUs in Yunnan Province and subtypes 6a and 6n may have originated in Vietnam and Myanmar, respectively.

## Introduction

Hepatitis C virus (HCV) is considered a principal cause of chronic liver disease, such as liver fibrosis, liver cirrhosis, and hepatocellular carcinoma, and currently infects about 170 million people worldwide [1], [2]. HCV has a positive-sense RNA genome and belongs to the genus *Hepacivirus* of the family *Flaviviridae*. At present, HCV is only known to naturally infect humans [3]. Individuals at high-risk for HCV infection include and percutaneous and intravenous drug users (IDUs), those receiving transfusion of blood products and hemodialysis, as well as those who engage in unprotected sex with multiple sex partners [4]. In China, intravenous drug use has become the predominant mode of HCV transmission [5], and HCV genotypes 3 and 6 are the most predominant genotypes and account for almost 70% of infections among IDUs [6]–[12].

Yunnan Province is located in southwestern China and borders on one of the world's most important poppy (*Papaver somniferum*) growing areas and opium producing bases (the ‘Golden Triangle,’ an area near the border of Laos, Myanmar and Thailand), and has been an important transfer station and drug trafficking route from the Golden Triangle to Yunnan Province and then to other regions of China. In 2012, Zhou et al. reported the prevalence of HIV, HCV, HBV and co-infection among IDUs. They also found the association of these viruses with high risk intravenous drug use behaviors in Yunnan Province and observed that a high proportion (77.7%) of IDUs were HCV-positive. [13]

In 2008, Xia et al. [8] investigated the distribution of HCV genotypes and subtypes among IDUs in Yunnan Province, and found that HCV genotype 3 was most predominant, followed by genotypes 6 and 1. In the present study, we assessed changes in HCV genotypes and subtypes among IDUs in Yunnan Province during the last 5 years and determined which HCV genotypes and subtypes were currently predominant among IDUs in Yunnan Province. Our results provided insights into HCV distribution patterns among IDUs in Yunnan Province and HCV transmission to surrounding regions. In addition, we identified changes in HCV genotype and subtype distribution patterns during the past 5 years in Yunnan Province.

## Materials and Methods

### Ethics statement

All participants submitted written informed consent to participate in the present study. The protocol was in accordance with the Helsinki Declaration and was approved by the Institutional Review Boards of the Institute of Medical Biology, Chinese Academy of Medical Sciences & Peking Union Medical College, and Kunming Center for Disease Control and Prevention.

### Samples

Samples were collected from 100 IDUs who were positive for HCV RNA and were enrolled at a drug rehabilitation center in Yunnan Province from January to May 2012. Whole blood samples (about 5 mL) were collected and immediately transported to the Kunming Center for Disease Control and Prevention (Kunming CDC) for detection of HCV antibodies and RNA.

### Serological assays

HCV antibody analysis was performed at the Kunming CDC. The presence of HCV antibodies was determined using an enzyme immunoassay kit (Wantai Inc., Beijing, China and Zhuhai Livzon, Inc., Zhuhai, China).

### RNA extraction and reverse transcription polymerase chain reaction ( RT-PCR) amplification

Total RNA was extracted from 200 uL of HCV-positive plasma using the MiniBEST Viral RNA/DNA Extraction Kit Ver 4.0 (TaKaRa Biotechnology, Co., Ltd., Dalian, China) and complementary DNA (cDNA) was synthesized using the PrimeScript 1st Strand cDNA Synthesis Kit (TaKaRa Biotechnology, Co., Ltd, Dalian, China.). As reported previously [14], the PCR primers to amplify the C/E1 regions of the HCV genome were W195 (5′-TTCATCATCATRTCCCANGCCAT-3′) and W196 (5′-AAYYTDCCCGGTTGCTCTTTYTCTAT-3′). Each 25-µL reaction volume contained 3 µL of cDNA as a template, 2.5 µL of 10× ExTaq PCR buffer (TaKaRa Biotechnology, Co., Ltd, Dalian, China.), 200 µM of each dNTP, 0.2 µM of each primer, and 0.625 U of ExTaq polymerase. DNA amplification consisted of 40 cycles at 54°C for 30 s, 72°C for 60 s, and 94°C for 30 s, followed by a 5-min extension step at 72°C.

### DNA purification, T-A cloning, and sequencing

Amplicons were purified using the Universal DNA Purification kit (Tiangen Biotech Co., Ltd., Beijing, China) and then cloned using the pMD19-T vector (TaKaRa Biotechnology Co. Ltd, Dalian, China.). Positive plasmids were sequenced by the Beijing Genomics Institute (Beijing, China).

### HCV genotyping and phylogenetic analyses

The sequences of all samples were aligned with HCV subtype reference sequences (C/E1 region) using the ClustalW multiple sequence alignment tool included in the Molecular Evolutionary Genetics Analysis (MEGA) v5.0 software package [15]. Reference HCV subtype sequences were downloaded from GenBank (accession numbers: EF115781.1, EF115784.1, EF115789.1, EF115797.1, GQ379231.1, JX102829.1, FJ687119.1, AY231591.1, JF721232.1, JF721240.1, JX102720.1, FJ210675.1, GQ206071.1, JF721261.1, AY739386.1, JX183345.1, JF721060.1, GQ205894.1, JF721029.1, JX183344.1, JF721079.1, GQ206083.1, AY739416.1, JQ303368.1, EU081433.1, FJ435090.1, and JQ318377.1). Phylogenetic trees of the HCV C/E1 regions were constructed using MEGA 5.0 software with the neighbor-joining method [15], and the reliability of the trees were evaluated using the bootstrap method with 1,000 replications

### Statistical analysis

**Figure 1:**
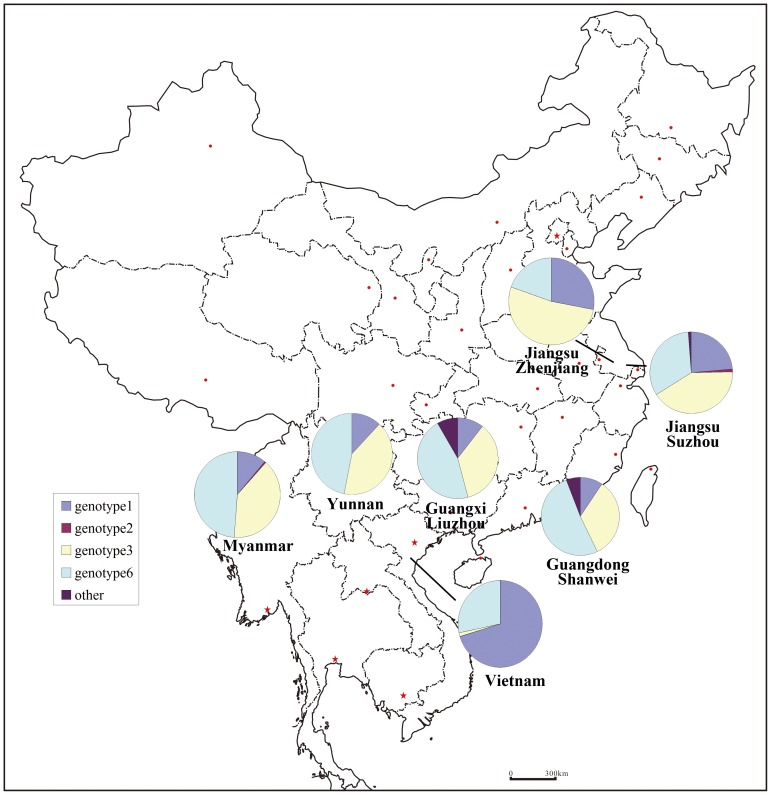
A map of Yunnan Province and other regions and HCV corresponding HCV distribution patterns.

HCV genotype and subtype frequencies for the present study were directly calculated and those from samples collected from IDUs from other regions were obtained from previous studies, as follows: Jiangsu Province [6], [12], Guangxi Province [9], Guangdong Province [11], Myanmar [10], and Vietnam [16]. The geographic distribution of IDUs from Yunnan Province and other regions used for comparison is shown in Figure 1. Jiangsu Province is located in eastern China, whereas Guangxi and Guangdong Provinces are located in southern China. Myanmar and Vietnam are located in Southeast Asia and border Guangxi and Yunnan Provinces. Significant differences between the HCV genotype frequencies among different regions were determined by a contingency test using SPSS software (version 13.0; SPSS, Inc., Chicago, IL, USA). A probability (*p*) value<0.05 was considered statistically significant.

## Results

### Prevalent HCV subtypes among IDUs in Yunnan Province

**Figure 2:**
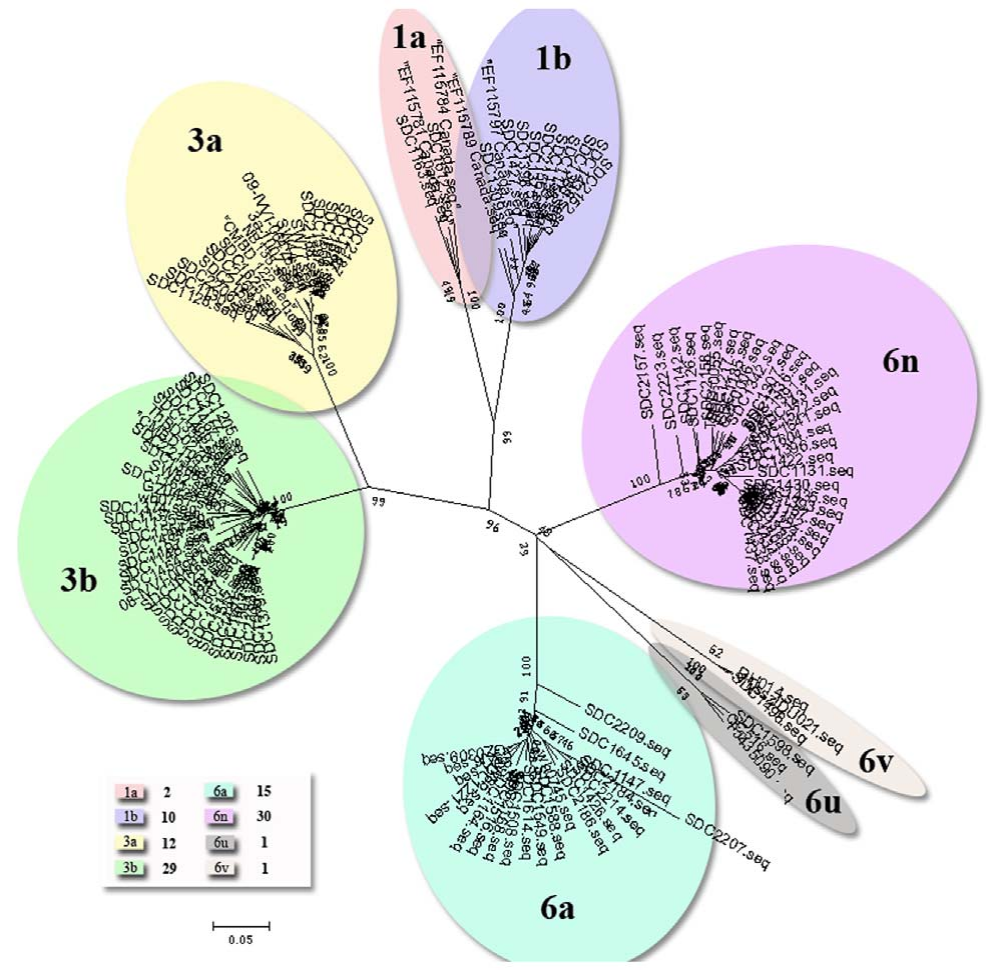
A phylogenetic tree estimated from C/E1 fragment sequences corresponding to H77 (nucleotide positions, 843–1315).

HCV C/E1 fragments were amplified successfully from all HCV-positive samples. In the present study, three HCV genotypes (1, 3 and 6) were identified, which included eight HCV subtypes (1a, 1b, 3a, 3b, 6a, 6n, 6u and 6v). HCV genotype 6 was the most predominant with a frequency of 47%, followed by genotype 3 (41%) and genotype 1 (12%). For HCV genotype 6, four subtypes were observed, whose frequencies were 15% (subtype 6a), 30% (subtype 6n) and 1% (subtype 6u and 6v, respectively). HCV genotype 3 included two subtypes, 3a (frequency, 12%) and 3b (frequency, 29%). HCV genotype 1 also included two subtypes, 1a (frequency, 2%) and 1b (frequency, 10%), respectively. A phylogenetic tree showing the relationship of the HCV C/E1 fragments is shown in Figure 2.

### HCV genotype distribution among IDUs from Yunnan Province and other regions

The frequencies of HCV genotypes among IDUs from different regions were showed in Table 1 and Figure 1. HCV genotype 6 was the most prevalent genotype in Yunnan (frequency, 47%), Guangxi (frequency, 46%) and Guangdong (frequency, 52%) Provinces, which are all located in southern or southwestern China. HCV genotype 6 was also common in Myanmar (frequency, 49%). There were no significant differences among the frequencies of HCV genotypes 1 and 3 in Yunnan, Guangxi, and Guangdong Provinces and Myanmar (*p*>0.05). However, there were significant differences among HCV genotypes 1 and 6 between Yunnan and Jiangsu Provinces, which are located in eastern China (*p*<0.05). There were no significant differences in the distribution of HCV genotype 3 between Yunnan and Jiangsu Provinces (*p*>0.05). Interestingly, the distribution of all three HCV genotypes showed statistically significant differences between Yunnan Province and Vietnam (*p*<0.05), despite their shared border.

**Table 1:** The frequencies (%) of HCV genotypes and subtypes within IDUs in current study and comparison with other in different regions.

	Yunnan	Yunnan	Guangxi	Guangdong	Jiangsu	Jiangsu	Vietnam	Myanmar
	(n = 100)	(n = 80)	Liuzhou(n = 96)	Shanwei(n = 136)	Suzhou(n = 85)	Zhenjiang(n = 71)	(n = 124)	(n = 145)
Genotype 1	12	21.2	11	10	24	28	70	11
Subtype 1a	2	1.2	7	0	4	1	42	4.1
Subtype1b	10	20	4	10	20	27	28	6.9
Genotype 2	0	0	0	0	10	0	0	0.7
Subtype 2a	0	0	0	0	1	0	0	0.7
Genotype 3	41	53.8	35	33	41	52	2	39.3
Subtype 3a	12	23.8	20	31	14	38	1	9.6
Subtype 3b	29	30	15	2	27	14	1	29.7
Genotype 6	47	25	46	52	33	20	28	49
Subtype 6a	15	5	46	52	22	8.5	22.5	0
Subtype 6n	30	11.2	0	0	10	3	0	38.6
Subtype 6u	1	8.8	0	0	1	0	0	0
Subtype 6v	1	0	0	0	0	0	0	0
Subtype 6e	0	0	0	0	0	8.5	4	0
Subtype 6 h	0	0	0	0	0	0	1.5	9
Others	0	0	8	5	1	0	0	1.4
References	This study	Xia *et al.*	Tan *et al.*	Fu *et al.*	Du *et al.*	Zhang *et al.*	Dunford *et al.*	Lwin *et al.*

Note: the other means other genotype 6 and dual infection.

### Distribution of HCV genotypes and subtypes among IDUs in the current study and a previous study conducted in Yunnan Province

In 2008, Xia et al. [8] investigated the distribution of HCV genotypes and subtypes among IDUs from Yunnan Province and found that genotype 3 was most predominant, followed by genotype 6 and genotype 1 (frequencies, 53.8%, 25% and 21.2%, respectively). Compared to the study by Xia et al., which assessed HCV genotypes 1, 3 and 6, the frequency of HCV genotype 6 significantly increased from 25% to 47% (*p*<0.05) (Table 1). HCV subtype analysis showed changes in HCV subtype frequencies: 1a, 1.2% to 2%; 1b, 20% to 10%; 3a, 23.8% to 12%; 3b, 30% to 29%; 6a, 5% to 15%; 6n, 11.2% to 30%; and 6u, 8.8% to 1%. In the present study, subtypes 3a, 6a, 6n and 6u showed different distribution patterns compared to a previous study (*p*<0.05).

## Discussion

Recently, several studies have reported an increase in HCV-positive IDUs [17]. In China, at least three genotypes (1, 3 and 6) and 11 subtypes (1a, 1b, 2a, 2b, 3a, 3b, 6a, 6n, 6u, 6v and 6e) were observed among IDUs [17]. However, the distribution of HCV genotypes and subtypes among IDUs differed between regions [6], [9]–[12], [16], [18]. Many studies have shown that the distribution of HCV was similar to that found along the drug-trafficking routes [6], [11], [17], [19] In the present study, we investigated the prevalence of HCV genotypes and subtypes in 100 IDUs from Yunnan Province and confirmed the circulation of three HCV genotypes (genotype 1, 3 and 6) and eight HCV subtypes (subtype 1a, 1b, 3a, 3b, 6a, 6n, 6u and 6v) (Tables 1).

A comparison of the HCV genotype and subtype distribution patterns between our results and those of other studies showed differences between eastern and southern and southwestern China. For example, HCV genotype 1 was most prevalent in Jiangsu Province (frequencies, 24% and 28%, respectively); however, its frequency was only 10% and 11% in southern and southwestern China, respectively. In contrast, HCV genotype 6 was more frequent in Yunnan Province and southern China (47% and 52%, respectively) than eastern China (20% and 33%, respectively). Next, we compared our results with those reported from Vietnam and Myanmar, which both border Yunnan Province, and found significant differences between Vietnam and Yunnan Province (*p*<0.05) in the frequencies of genotype 1 (70% vs. 12%, respectively), genotype 3 (2% vs. 41%, respectively), and genotype 6 (28% vs. 47%, respectively). In contrast, HCV distribution in Myanmar was similar to that in Yunnan Province and other southern and southwestern regions in China, including Guangxi and Guangdong Provinces (*p*>0.05).

HCV genotype analysis showed that HCV genotype 6 was predominant in southern and southwestern regions of China (46% to 52%), although frequencies of other HCV of subtype 6 were different among these regions. For example, the distribution frequency of HCV subtype 6a was significantly greater in Guangxi and Guangdong Provinces compared to Yunnan Province (46% and 52% vs. 15%; *p*<0.05). In 2012, Fu et al. [11] proposed that HCV subtype 6a originated in Vietnam and circulated to neighboring regions, including Guangxi and Yunnan Provinces. The different distribution frequencies of subtype 6a between Guangxi and Yunnan Provinces may be explained by the drug trafficking from Vietnam to Yunnan Province by way of Guangxi Province, which has been identified as an important drug transfer region. Surprisingly, HCV subtype 6a was not detected in samples from Myanmar. In 2009, Pybus et al. demonstrated that HCV subtype 6n may have originated in Thailand/Myanmar and has since become predominant in Yunnan Province and Myanmar [20], but was not detected in Guangxi and Guangdong Provinces or Vietnam. The different distribution patterns of HCV subtypes 6a and 6n between Yunnan Province, Myanmar, and Vietnam indicated that they may have originated in Vietnam and Myanmar, respectively. HCV subtype 3b was also a common HCV subtype in Yunnan Province. Its frequency in Yunnan Province (29%) was similar to that in Myanmar (29.7%), but much higher than in Vietnam (1%). The HCV subtype 3b may spread to other eastern and southern regions of China, such as Jiangsu (14% and 27%) and Guangxi Provinces (15%), via drug trafficking from Yunnan Province and/or Myanmar, but not Vietnam [6], [9], [10], [12], [16].

A comparison of our results with those of Xia [8] in 2008 showed no significant changes in the distribution of HCV genotypes over the past 5 years. However, the frequency of HCV subtypes 1b and 3a decreased from 20% to 10% and 23.8% to 12%, respectively, whereas the frequency of HCV subtypes 6a and 6n increased from 5% to 15% and 11.2% to 30%, respectively. These results indicated that the increased frequency of HCV subtypes 6a and 6n may have circulated along drug trafficking routes from Myanmar and Vietnam and fueled the increased distribution of subtype 6 among IDUs in Yunnan Province.

Since the sample size of the IDUs in the present study was relatively small, we were unable to provide a more comprehensive scenario of HCV transmission in Yunnan Province. However, our results provided further information to support the assertion that this drug trafficking route has influenced the rate of HCV transmission and changes in genotypes among IDUs in Yunnan Province.
